# Preoperative arterial lactate and outcome after surgery for type A aortic dissection: The ERTAAD multicenter study^☆^

**DOI:** 10.1016/j.heliyon.2023.e20702

**Published:** 2023-10-05

**Authors:** Fausto Biancari, Francesco Nappi, Giuseppe Gatti, Andrea Perrotti, Amélie Hervé, Stefano Rosato, Paola D'Errigo, Matteo Pettinari, Sven Peterss, Joscha Buech, Tatu Juvonen, Mikko Jormalainen, Caius Mustonen, Till Demal, Lenard Conradi, Marek Pol, Petr Kacer, Angelo M. Dell’Aquila, Konrad Wisniewski, Igor Vendramin, Daniela Piani, Luisa Ferrante, Timo Mäkikallio, Eduard Quintana, Robert Pruna-Guillen, Antonio Fiore, Thierry Folliguet, Giovanni Mariscalco, Metesh Acharya, Mark Field, Manoj Kuduvalli, Francesco Onorati, Cecilia Rossetti, Sebastien Gerelli, Dario Di Perna, Enzo Mazzaro, Angel G. Pinto, Javier Rodriguez Lega, Mauro Rinaldi

**Affiliations:** a Heart and Lung Center, Helsinki University Hospital, University of Helsinki, Helsinki, Finland; b Department of Medicine, South-Karelia Central Hospital, University of Helsinki, Lappeenranta, Finland; c Department of Cardiac Surgery, Centre Cardiologique du Nord de Saint-Denis, Paris, France; d Division of Cardiac Surgery, Cardio-thoracic and Vascular Department, Azienda Sanitaria Universitaria Giuliano Isontina, Trieste, Italy; e Department of Thoracic and Cardiovascular Surgery, University of Franche-Comte, Besancon, France; f Center for Global Health, National Health Institute, Rome, Italy; g Department of Cardiac Surgery, Ziekenhuis Oost Limburg, Genk, Belgium; h LMU University Hospital, Ludwig Maximilian University, Munich, Germany; i German Centre for Cardiovascular Research, Partner Site Munich Heart Alliance, Munich, Germany; j Research Unit of Surgery, Anesthesia and Critical Care, University of Oulu, Oulu, Finland; k Department of Cardiovascular Surgery, University Heart & Vascular Center Hamburg, Hamburg, Germany; l Department of Cardiac Surgery, Third Faculty of Medicine, Charles University and University Hospital Kralovske Vinohrady, Prague, Czech Republic; m Department of Cardiothoracic Surgery, University Hospital Muenster, Muenster, Germany; n Cardiothoracic Department, University Hospital, Udine, Italy; o Cardiac Surgery, Molinette Hospital, University of Turin, Turin, Italy; p Department of Cardiovascular Surgery, Hospital Clínic de Barcelona, University of Barcelona, Spain; q Department of Cardiac Surgery, Hôpitaux Universitaires Henri Mondor, Assistance Publique-Hôpitaux de Paris, Creteil, France; r Department of Cardiac Surgery, Glenfield Hospital, Leicester, United Kingdom; s Liverpool Centre for Cardiovascular Sciences, Liverpool Heart and Chest Hospital, Liverpool, United Kingdom; t Division of Cardiac Surgery, University of Verona Medical School, Verona, Italy; u Centre Hospitalier Annecy Genevois, France; v Cardiovascular Surgery Department, University Hospital Gregorio Marañón, Madrid, Spain

**Keywords:** Type A aortic dissection, Aortic dissection, Lactic acid, Arterial lactate, Hyperlactatemia

## Abstract

**Background:**

Acute type A aortic dissection (TAAD) is associated with significant mortality and morbidity. In this study we evaluated the prognostic significance of preoperative arterial lactate concentration on the outcome after surgery for TAAD.

**Methods:**

The ERTAAD registry included consecutive patients who underwent surgery for acute type A aortic dissection (TAAD) at 18 European centers of cardiac surgery.

**Results:**

Data on arterial lactate concentration immediately before surgery were available in 2798 (71.7 %) patients. Preoperative concentration of arterial lactate was an independent predictor of in-hospital mortality (mean, 3.5 ± 3.2 vs 2.1 ± 1.8 mmol/L, adjusted OR 1.181, 95%CI 1.129–1.235). The best cutoff value preoperative arterial lactate concentration was 1.8 mmol/L (in-hospital mortality, 12.0 %, vs. 26.6 %, p < 0.0001). The rates of in-hospital mortality increased along increasing quintiles of arterial lactate and it was 12.1 % in the lowest quintile and 33.6 % in the highest quintile (p < 0.0001). The difference between multivariable models with and without preoperative arterial lactate was statistically significant (p = 0.0002). The NRI was 0.296 (95%CI 0.200–0.391) (p < 0.0001) with −17 % of events correctly reclassified (p = 0.0002) and 46 % of non-events correctly reclassified (p < 0.0001). The IDI was 0.025 (95%CI 0.016–0.034) (p < 0.0001). Six studies from a systematic review plus the present one provided data for a pooled analysis which showed that the mean difference of preoperative arterial lactate between 30-day/in-hospital deaths and survivors was 1.85 mmol/L (95%CI 1.22–2.47, p < 0.0001, I^2^ 64 %).

**Conclusions:**

Hyperlactatemia significantly increased the risk of mortality after surgery for acute TAAD and should be considered in the clinical assessment of these critically ill patients.

Key-words: Aortic dissection; Lactate; Acidosis.

## 1 Introduction

Emergency surgical repair is a salvage procedure for patients with type A aortic dissection (TAAD), still surgery is associated with significant mortality [1,2]. TAAD is frequently associated with systemic or end-organ hypoperfusion, which significantly decrease the chances to survive surgery [3,4]. Arterial lactate is a widely recognized reliable marker of reduced oxygen delivery in cardiovascular diseases [[5], [6], [7]]. There is evidence that arterial lactate may have prognostic importance also in patients with TAAD [9,10]. Beside its prognostic significance, this biomarker may be potentially useful to guide the optimal timing of surgery before severe metabolic derangements subside. Furthermore, in patients with hemodynamic instability often it is not feasible to gather reliable information on patient's risk factors and symptoms of visceral and peripheral malperfusion. Instead, arterial lactate may provide an objective measure of the magnitude of reduced end-organ oxygen delivery. The present European retrospective, multicenter study aimed to evaluate prognostic significance of preoperative arterial lactate on the outcome of patients after surgery for acute TAAD.

## 2 Methods

### 2.1 Study population

The study population is a consecutive series of patients who underwent surgery for acute TAAD and whose data were retrospectively collected in to the European Registry of Type A Aortic Dissection (ERTAAD). These patients were operated at 18 hospitals in eight European countries (Belgium, Czech Republic, Finland, France, Germany, Italy, Spain and the United Kingdom) from January 1, 2005 to March 31, 2021. This study received approval from the Ethical Review Board of the Helsinki University Hospital, Finland (April 21, 2021, diary no. HUS/237/2021) as well as from the Ethical Review Board of each participating hospital. The informed consent of participating patients was waived because of the retrospective nature of this study. Data on pre-specified baseline, operative and outcome variables were collected in to a Microsoft Access datasheet (Redmond, Washington, USA). Data on the date of death were collected retrospectively from electronic national registries as well as by contacting regional hospitals, patients and their relatives. The study was conducted according to the Strengthening the Reporting of Observational Studies in Epidemiology (STROBE) guidelines [11].

### 2.2 Inclusion and exclusion criteria

The inclusion criteria of this study were the following: 1) patients with TAAD; 2) patients aged >18 years; 3) onset of symptoms related to TAAD within 7 days from surgery; 4) primary operation for acute TAAD; 5) any cardiac surgical procedure required during surgery for TAAD [8]. The exclusion criteria were the following: 1) age less than 18 years; 2) onset of symptoms >7 days from surgery; 3) prior surgery for TAAD; 4) TAAD of retrograde origin (with primary tear located in the descending aorta); 5) concomitant endocarditis; 6) TAAD due to blunt or penetrating chest trauma [10].

### 2.3 Clinical and laboratory variables

Information regarding the definition criteria of other risk factors, the stratification of the urgency of the procedure and interventions have been previously described [10]. Salvage was defined as a procedure performed in patients requiring cardiopulmonary resuscitation with external chest compression or open cardiac massage *en route* to the operating theatre or after anesthesia induction [10].

Baseline concentration of arterial lactate and other biomarkers were obtained immediately before the start of surgery. Furthermore, we collected data on peak concentration of arterial lactate after surgery to evaluate the impact of baseline concentration of arterial lactate on its postoperative clearance.

### 2.4 Study outcomes

In-hospital mortality was the primary outcome of the study and refers to death from any cause occurring during the index hospitalization. Secondary outcomes were stroke/global brain ischemia, paraplegia/paraparesis, mesenteric ischemia, sepsis, dialysis, reoperation for intrathoracic bleeding, heart failure, need of mechanical circulatory support (use of intra-aortic balloon pump and/or extracorporeal membrane oxygenation), upper or lower limb ischemia, major lower limb amputation, surgery for intestinal complications and late mortality. A composite outcome including in-hospital death and/or stroke/global brain ischemia was also a secondary outcome. Late mortality was defined as all-cause death during the index hospitalization and follow-up period. These outcomes were defined according to previously reported criteria [10].

### 2.5 Meta-analysis

On December 5th, 2022 a systematic review of the literature was performed through PubMed, Scopus and Google Scholar. Studies in English language published on preoperative concentration of arterial lactate before surgery for TAAD were included in this analysis. Abstracts were not included in this analysis. The search was performed using the words “lactate” and “aortic dissection”. Articles were retrieved and checked by two authors (F.B., G.M.).

### 2.6 Statistical analysis

Continuous variables were reported as means and standard deviations. Categorical variables were reported as counts and percentages. The Mann-Whitney's test was used to compare continuous variables. Tests of between-subjects effects was performed to evaluate the impact of preoperative and postoperative peak arterial lactate on in-hospital mortality. The chi-square test and Fisher's exact test were used for categorical variables. The best cutoff value for continuous variables was identified using the Liu method. Classification and regression tree analysis was performed to evaluate the prognostic impact of arterial lactate and age on the risk in-hospital mortality. Gini's method with minimum change improvement of 0.0001, parent node 100 and child node 50. Multilevel mixed-effects logistic regression and logistic regression were used for identification of independent risk factors of in-hospital mortality by including covariates with p < 0.20 in univariable analysis. Discrimination of the regression model as well as prognostic ability of preoperative arterial lactate was evaluated by calculating the area under the receiver operating characteristics curve (ROC), while calibration was evaluated with the Hosmer-Lemeshow's test. Differences between ROC curves were assessed with the DeLong's test. The improvement of discrimination of the regression model including preoperative arterial lactate as compared to the regression model without it was estimated by calculating the net reclassification index (NRI) and integrated discrimination improvement (IDI). Predictors of preoperative arterial lactate concentration were identified by linear regression with the stepwise backward method. Meta-analysis was conducted to investigate the differences between in-hospital deaths and survivors after surgery for TAAD in terms of preoperative arterial lactate concentration. This analysis was performed using the random-effects method since heterogeneity was anticipated. The mean and standard deviation were extracted from the median and interquartile ranges using the method proposed by Wan et al. [12]. P < 0.05 was set for statistical significance. Statistical analyses were performed with the SPSS (version 27.0, SPSS Inc., IBM, Chicago, Illinois, USA), Stata (version 15.1, StataCorp LLC, College Station, Texas, USA) and RevMan 5.4.1 (the Cochrane Collaboration, 2020) statistical softwares.

## 3 Results

### 3.1 Preoperative arterial lactate concentration and outcome

**Fig. 1:**
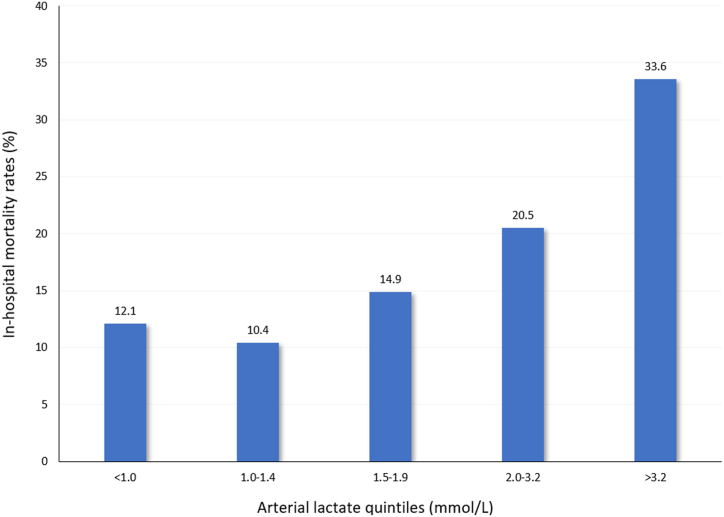
In-hospital mortality rates along increasing quintiles of preoperative arterial lactate concentrations in patients operated for TAAD (p < 0.0001).

**Fig. 2:**
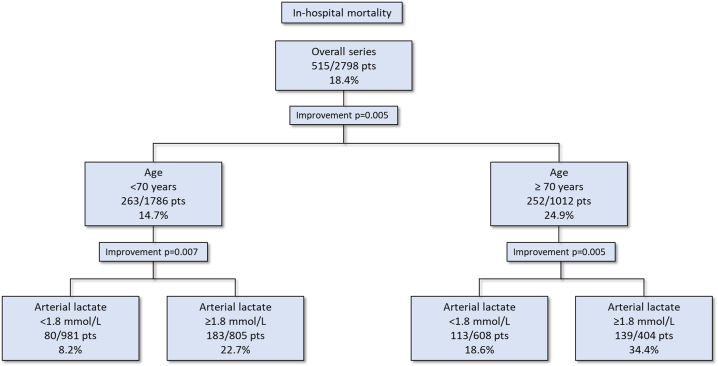
Classification and regression tree summarizing the in-hospital mortality in TAAD patients according to a cutoff value of 70 years for age and 1.8 mmol/L of arterial lactate.

Among 3902 consecutive patients, data on preoperative arterial lactate concentration was available in 2798 (71.7 %) patients. In-hospital mortality of these patients was 18.4 % and baseline and operative data of survivors and deaths are summarized in Table 1. Predictors of in-hospital mortality in univariate analysis are listed in Table 1. Preoperative concentration of arterial lactate was higher among patients who died during the index hospitalization (mean, 3.5 ± 3.2 vs 2.1 ± 1.8 mmol/L, p < 0.0001). The rates of in-hospital mortality increased along increasing quintiles of arterial lactate (p < 0.0001) and it was 12.1 % in the lowest quintile and 33.6 % in the highest quintile (Fig. 1). The best cutoff value preoperative arterial lactate concentration was 1.8 mmol/L (In hospital mortality: ≤1.8 mmol/L,12.0 % vs. >1.8 mmol/L, 26.6 %, p < 0.0001; sensitivity 0.67, specificity 0.59). The prognostic effect of age cutoff value of 70 years and of arterial lactate concentration of 1.8 mmol/L is shown in Fig. 2. In-hospital mortality in patients ≥70 years with preoperative arterial lactate ≥1.8 mmol/L was as high as 34.4 %. On the contrary in-hospital mortality in patients <70 years with preoperative arterial lactate <1.8 mmol/L was as low as 8.2 %.

**Table 1:** Patients’ characteristics and operative data of TAAD patients and predictors of in-hospital mortality.

	Survivor N = 2283	In-hospital death N = 515	Univariable analysis P-value	Multivariable analysis without arterial lactate OR, 95%CI	Multivariable analysis with arterial lactate OR, 95%CI
Age, years	62.7 (12.9)	67.3 (12.7)	<0.0001	1.030, 1.020–1.039	1.033, 1.023–1.043
Females, No. (%)	693 (30.4)	164 (31.8)	0.508		
eGFR, mL/min/1.73 m^2^	72 [23]	60 [22]	<0.0001	0.981, 0.976–0.986	0.985, 0.979–0.990
Arterial lactate, mmol/L	2.1 (1.8)	3.5 (3.2)	<0.0001		1.181, 1.129–1.235
Genetic syndrome, No. (%)	49 (2.1)	5 (1.0)	0.108		
Bicuspid aortic valve, No. (%)	89 (3.9)	15 (2.9)	0.289		
Iatrogenic dissection, No. (%)	50 (2.2)	22 (4.3)	0.007	2.027, 1.139–3.606	2.135, 1.191–3.826
Diabetes, No. (%)	104 (4.6)	35 (6.8)	0.035		
Stroke, No. (%)	89 (3.9)	24 (4.7)	0.457		
Pulmonary disease, No. (%)	188 (8.2)	48 (9.3)	0.423		
Extracardiac arteriopathy, No. (%)	122 (5.3)	49 (9.5)	<0.0001		
Prior cardiac surgery, No. (%)	73 (3.2)	17 (3.3)	0.904		
Cardiac massage, No. (%)	65 (2.8)	66 (12.8)	<0.0001		
Shock requiring inotropes, No. (%)	371 (16.3)	153 (29.7)	<0.0001		
Cerebral malperfusion, No. (%)	488 (21.4)	175 (34.0)	<0.0001	1.632, 1.292–2.062	1.560, 1.229–1.979
Spinal malperfusion, No. (%)	43 (1.9)	15 (2.9)	0.139		
Renal malperfusion, No. (%)	199 (8.7)	70 (13.6)	0.001		
Mesenteric malperfusion, No. (%)	77 (3.4)	47 (9.1)	<0.0001	2.178, 1.416–3.350	1.815, 1.169–2.818
Peripheral malperfusion, No. (%)	337 (14.8)	115 (22.3)	<0.0001	1.662, 1.269–2.178	1.551, 1.178–2.042
DeBakey type I dissection, No. (%)	1990 (87.2)	435 (84.5)	0.265		
Penn classification, No. (%)			<0.0001		
a	1320 (57.8)	182 (35.3)			
b	583 (25.5)	162 (31.5)			
c	134 (5.9)	61 (11.8)			
b + c	246 (10.8)	110 (21.4)			
Operative data					
Salvage procedure, No. (%)	70 (3.1)	70 (13.6)	<0.0001	4.185, 2.834–6.181	3.111, 2.070–4.675
Partial or total arch replacement, No. (%)	402 (17.6)	115 (22.3)	0.013	1.323, 1.002–1.747	1.356, 1.023–1.798
Aortic root replacement No. (%)	634 (27.8)	153 (29.7)	0.377		
Coronary artery bypass grafting, No. (%)	175 (7.7)	84 (16.3)	<0.0001	2.092, 1.506–2.908	2.093, 1.500–2.920
XCT time, mean (SD), min	119 (54)	132 (65	<0.0001	1.005, 1.003–1.007	1.005, 1.003–1.007

Continuous variables are reported as mean and standard deviation. Categorical variables are reported as counts and standard deviation. eGFR = estimated glomerular filtration rate according to the CKD-EPI equation; XCT = aortic cross clamping time.

### 3.2 Risk factors of in-hospital mortality

A logistic regression model not including preoperative arterial lactate identified independent risk factors for in-hospital mortality (Hosmer-Lemeshow, p = 0.065) with an area under the ROC curve of 0.740 (95%CI 0.716–0.764) (Table 1). Logistic regression with a model including preoperative arterial lactate, identified independent risk factors for in-hospital mortality along with arterial lactate (OR 1.181, 95%CI 1.129–1.235) (Hosmer-Lemeshow, p = 0.666) with an area under the ROC curve of 0.759 (95%CI 0.736–0.782) (Table 1). The difference between the areas under the ROC curve of these regression model was statistically significant (p = 0.0002). The NRI was 0.296 (95%CI 0.200–0.391) (p < 0.0001) with −17 % of events correctly reclassified (p = 0.0002) and 46 % of non-events correctly reclassified (p < 0.0001). The IDI was 0.025 (95%CI 0.016–0.034) (p < 0.0001).

When multilevel mixed-effects logistic regression was performed, preoperative arterial lactate was confirmed being an independent predictor of in-hospital mortality (OR 1.179, 9%CI 1122-1.239) with a similar increased in the area under the ROC curve (0.799, 95%CI 0.669–0.820 vs. 0.813, 95%CI 0.792–0.833, p = 0.0005).

In univariable analysis, preoperative concentration of arterial lactate was associated also with significantly increased risk of all secondary outcomes (Table 2). It is worth noting the rather large area under the ROC curve in predicting postoperative heart failure and need of mechanical circulatory support.

**Table 2:** Predictive ability of preoperative arterial lactate concentration in predicting early adverse events.

Outcomes	No. (%)	P-value	ROC analysis AUC, 95%CI
Hospital mortality, No. (%)	515 (18.4)	<0.0001	0.652, 0.624–0.679
Stroke/global brain ischemia, No. (%)	523 (18.7)	<0.0001	0.604, 0.577–0.631
Composite outcome, No. (%)	835 (29.8)	<0.0001	0.647, 0.625–0.670
Paraparesis/paraplegia, No. (%)	161 (5.8)	0.046	0.547, 0.505–0.589
Mesenteric ischemia, No. (%)	127 (4.5)	<0.0001	0.661, 0.611–0.710
Sepsis, No. (%)	415 (14.8)	<0.0001	0.554, 0.526–0.582
Dialysis, No. (%)	431 (15.4)	<0.0001	0.595, 0.566–0.624
Reoperation for bleeding, No. (%)	391 (14.0)	0.025	0.535, 0.504–0.566
Heart failure, No. (%)	442 (15.8)	<0.0001	0.610, 0.581–0.639
Mechanical circulatory support, No. (%)	108 (3.9)	<0.0001	0.643, 0.590–0.696
Acute lower limb ischemia, No. (%)	101 (3.6)	<0.0001	0.543, 0.588–0.699
Major lower limb amputation, No. (%)	14 (0.5)	<0.0001	0.619, 0.434–0.805
Acute upper limb ischemia, No. (%)	12 (0.4)	<0.0001	0.478, 0.342–0.615
Surgery for intestinal complications, No. (%)	17 (0.6)	<0.0001	0.748, 0.636–0.860

### 3.3 Risk factors associated with increased preoperative arterial lactate concentration

Univariable analysis showed that female gender, estimated glomerular filtration rate, salvage procedure, shock requiring inotropes, cardiac massage and malperfusion were associated with increased preoperative concentration of arterial lactate (Table 3). Linear regression showed that salvage procedure (coefficient 1.694, 95%CI 1.310–2.078) and shock requiring inotropes (coefficient 1.301, 95%CI 0.827–1.256) were the only independent predictors of hyperlactatemia.

**Table 3:** Patients’ characteristics and operative data of TAAD patients predictive of preoperative arterial lactate concentration in univariable and multivariable analyses.

	Univariable analysis P-value	Multivariable analysis Coefficient, 95%CI
Age, years	0.301	
Females, No. (%)	0.007	
eGFR, mL/min/1.73 m^2^	<0.0001	
Genetic syndrome, No. (%)	0.143	
Bicuspid aortic valve, No. (%)	0.269	
Iatrogenic dissection, No. (%)	0.070	
Diabetes, No. (%)	0.805	
Stroke, No. (%)	0.385	
Pulmonary disease, No. (%)	0.032	
Extracardiac arteriopathy, No. (%)	0.148	
Prior cardiac surgery, No. (%)	0.062	
Cardiac massage, No. (%)	<0.0001	
Shock requiring inotropes, No. (%)	<0.0001	1.301, 0.827–1.256
Cerebral malperfusion, No. (%)	<0.0001	
Spinal malperfusion, No. (%)	<0.0001	
Renal malperfusion, No. (%)	<0.0001	
Mesenteric malperfusion, No. (%)	<0.0001	
Peripheral malperfusion, No. (%)	<0.0001	
DeBakey type I dissection, No. (%)	0.067	
Penn classification, No. (%)	<0.0001	
a		
b		
c		
b + c		
Operative data		
Salvage procedure, No. (%)	<0.0001	1.694, 1.310–2.078
Partial or total arch replacement, No. (%)	0.579	
Aortic root replacement No. (%)	0.493	
Coronary artery bypass grafting, No. (%)	0.298	
XCT time, mean (SD), min	0.469	

eGFR = estimated glomerular filtration rate according to the CKD-EPI equation; XCT = aortic cross clamping time.

When patients who underwent salvage procedure were excluded from the analysis, shock requiring inotropes (coefficient 0.955, 95%CI 0.740–1.170) and peripheral malperfusion (0.554, 95%CI 0.343–0.764) were independent predictors of preoperative concentration of arterial lactate.

### 3.4 Arterial lactate clearance

Data on postoperative peak lactate was available in 2692 (96.2 %) patients and it was significantly increased in patients who died during the index hospitalization (mean 9.9 ± 4.9 vs. 5.3 ± 3.8 mmol/L, p < 0.0001). Repeated measure test confirmed the prognostic importance of arterial lactate clearance on in-hospital mortality (P < 0.0001).

### 3.5 Risk factors of late mortality

Table 4 summarize the risk factors associated with late mortality. Preoperative arterial lactate was an independent risk factors for late mortality (HR 1.100, 95%CI 1.072–1.129) (Table 4). However, this effect was mainly related to the increased risk of in-hospital mortality, because after excluding hospital deaths from the analysis, preoperative arterial lactate was not an independent predictor of late mortality.

**Table 4:** Patients’ characteristics and operative data of TAAD patients predictive of late all-cause mortality in univariable and multivariable analyses.

	Univariable analysis P-value	Multivariable analysis HR, 95%CI
Age, years	<0.0001	1.038, 1.032–1.045
Females, No. (%)	0.001	
eGFR, mL/min/1.73 m^2^	<0.0001	0.989, 0.986–0.993
Arterial lactate, mmol/L	<0.0001	1.100, 1.072–1.129
Genetic syndrome, No. (%)	0.057	
Bicuspid aortic valve, No. (%)	0.002	
Iatrogenic dissection, No. (%)	0.015	
Diabetes, No. (%)	0.003	
Stroke, No. (%)	0.039	
Pulmonary disease, No. (%)	<0.0001	
Extracardiac arteriopathy, No. (%)	<0.0001	1.473, 1.182–1.836
Prior cardiac surgery, No. (%)	0.016	
Cardiac massage, No. (%)	<0.0001	
Shock requiring inotropes, No. (%)	<0.0001	
Cerebral malperfusion, No. (%)	<0.0001	1.307, 1.130–1.511
Spinal malperfusion, No. (%)	0.398	
Renal malperfusion, No. (%)	0.012	
Mesenteric malperfusion, No. (%)	<0.0001	
Peripheral malperfusion, No. (%)	<0.0001	1.236, 1.047–1.460
DeBakey type I dissection, No. (%)	0.161	
Penn classification, No. (%)	<0.0001	
a		
b		
c		
b + c		
Operative data		
Salvage procedure, No. (%)	<0.0001	2.028, 1.592–2.582
Partial or total arch replacement, No. (%)	0.001	1.396, 1.177–1.657
Aortic root replacement No. (%)	0.034	
Coronary artery bypass grafting, No. (%)	<0.0001	
XCT time, mean (SD), min	0.001	1.003, 1.002–1.005

eGFR = estimated glomerular filtration rate according to the CKD-EPI equation; HR = hazard ratio; XCT = aortic cross clamping time.

## 4 Results of meta-analysis

**Fig. 3:**
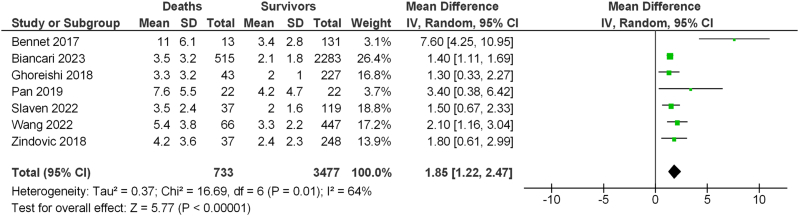
Forest plot of mean difference of preoperative concentrations of arterial lactate in seven clinical studies reporting on TAAD patients.

Literature search yielded 104 studies of potential interest. Six studies [8,[13], [14], [15], [16], [17]] plus the present one provided data for a pooled analysis which showed that the mean difference of preoperative arterial lactate between 30-day/in-hospital deaths and survivors was 1.85 mmol/L (95%CI 1.22–2.47, I^2^ 64 %) and was statistically significant (Fig. 3).

## 5 Discussion

The results of this study can be summarized as follows: 1) preoperative concentration of arterial lactate was predictive of in-hospital death after surgery for acute TAAD; 2) adding preoperative arterial lactate to other independent risk factors of in-hospital mortality significantly increased the predictive ability of regression model; 3) increased concentration of arterial lactate was associated with increased risk of in-hospital adverse events after surgery for acute TAAD.

This rather large series provided further evidence of the prognostic impact of hyperlactatemia in this critically ill patient population. Increased concentrations of arterial lactate have been shown also in other acute cardiac conditions [18,19], as a result primarily of decreased oxygen delivery [20], and the negative prognostic impact of hyperlactatemia might be secondary to cardiac failure [21]. Hyperlactatemia is a valuable marker of multiple end-organ injury related to other conditions [22,23]. In the present study, salvage procedure, i.e. surgery in patients requiring cardiac massage *en route* to the operating room or after anesthesia induction, and cardiogenic shock requiring inotropes, were independent predictors of hyperlactatemia. This confirms that an acute state of cardiogenic shock is the main cause of hyperlactatemia in TAAD patients. However, when patients undergoing salvage procedures were excluded from the analysis, peripheral malperfusion was also an independent predictor of increased levels of arterial lactate. Furthermore, malperfusion of any nature was associated with hyperlactatemia in univariate analysis. This means that in TAAD patients, regional hypoperfusion secondary to static and/or dynamic mechanisms of obstruction of aortic branch vessels may have an impact on the development of hyperlactatemia as well.

The heart has a metabolic flexibility and lactate becomes its preferred metabolic substrate once hyperlactatemia is established [24,25]. However, it is unclear to what extent lactate in heart diseases provides benefits [25]. Heart failure is associated with an increased amount of intracellular lactate in the myocardium which is preferably used alongside ketones as a source of energy [26]. Still, hyperlactatemia is associated with increased risk of mortality in acute heart failure [18]. The infusion of arterial lactate in the experimental setting has been shown to have a negative inotropic effect on the ventricle [[27], [28], [29]]. Similarly, ventricular function worsens with decreasing level of pH [27,29]. Depressed ventricular function associated with acidemia has been observed also in the clinical setting [30]. Therefore, hyperlactatemia and acidemia may have a negative effect on the recovery of the myocardium in TAAD patients. This may be the reason why in the present study, preoperative arterial lactate was associated with increased risk of postoperative heart failure and need of mechanical circulatory support in acute TAAD patients as shown by rather large areas under the ROC curves (Table 2). A study by Zhang et al. [31] demonstrated that increased preoperative arterial lactate can be predictor of end-organ injury such as acute kidney failure as well.

The prognostic impact of arterial lactate has been the topic of investigation of several studies, but many of them investigated the prognostic ability of postoperative hyperlactatemia [[32], [33], [34]], i.e. when perioperative hypoperfusion has led to significant, and often irreversible, metabolic derangements. However, also preoperative arterial lactate was found predictive of operative mortality in TAAD patients and were included in three specific risk scoring methods [14,35,36]. The prognostic importance of preoperative arterial lactate was further confirmed in the present pooled analyses of six available studies [8,[13], [14], [15], [16], [17]] (Fig. 3). It is worth noting that the mean difference between operative survivors and deaths observed in this pooled analysis was only 1.8 mmol/L. In this study the difference in preoperative arterial lactate between survivors and hospital deaths was rather limited but increased significantly after surgery as shown by rather high postoperative concentrations of arterial lactate in patients who died during the index hospitalization. This finding suggests also that even a modest increase in arterial lactate in these patients is associated with a critical systemic or end-organ hypoperfusion which should prompt immediate surgical repair.

The retrospective nature is the main limitation of this study. Second, we had data only on peak postoperative arterial lactate concentration. Therefore, a temporal analysis of clearance of arterial lactate is not feasible from this dataset. Third, interinstitutional differences in measurements of arterial lactate and/or timing of operation might have occurred, but analysis with multilevel mixed-effects logistic regression, considering any cluster effect, confirmed the findings of conventional logistic regression. Fourth, data on preoperative arterial lactate was available in 71.7 % of patients. We do not know whether missing data might have affected the present results. However, present data is from a relatively large study population and pooled analysis showed that preoperative arterial lactate was significantly higher among operative deaths in all studies gathered from systematic review. Finally, we do not have data on preoperative level of pH in these patients for a throughout analysis of the prognostic impact of. preoperative acidemia.

The strengths of the present analysis are the relatively large size of the study population and the multicenter nature of the study which makes the results generalizable. The present results confirmed the findings of a few previous studies of small size on acute TAAD patients and provided evidence also of its negative prognostic impact on major adverse postoperative events such as neurological complications, heart failure, mesenteric ischemia and need of abdominal surgical procedures.

In conclusion, the present study demonstrated that preoperative arterial lactate is predictive of in-hospital mortality and other early adverse postoperative events after surgery for acute TAAD. Our analysis showed that even a modest increase above normal levels of arterial lactate before surgery for TAAD significantly increases the mortality risk in these patients.

## Data availability statement

The authors do not have permission to share data.

## Funding

This study was not financially supported.

AUC = area under the curve; ROC = receiver operating characteristics curve; Composite outcome = death and/or stroke/global brain ischemia; Mechanical circulatory support = intra-aortic balloon pump and/or extracorporeal membrane oxygenation.

## CRediT authorship contribution statement

**Fausto Biancari:** Conceptualization, Data curation, Formal analysis, Investigation, Methodology, Project administration, Software, Supervision, Validation, Writing – original draft, Writing – review & editing. **Francesco Nappi:** Conceptualization, Data curation, Investigation, Writing – review & editing. **Giuseppe Gatti:** Conceptualization, Data curation, Investigation, Writing – review & editing. **Andrea Perrotti:** Conceptualization, Data curation, Investigation, Writing – review & editing. **Amélie Hervé:** Conceptualization, Data curation, Investigation, Writing – review & editing. **Stefano Rosato:** Formal analysis, Writing – review & editing. **Paola D'Errigo:** Formal analysis, Writing – review & editing. **Matteo Pettinari:** Conceptualization, Data curation, Investigation, Writing – review & editing. **Sven Peterss:** Conceptualization, Data curation, Investigation, Writing – review & editing. **Joscha Buech:** Conceptualization, Data curation, Investigation, Writing – review & editing. **Tatu Juvonen:** Conceptualization, Data curation, Investigation, Writing – review & editing. **Mikko Jormalainen:** Conceptualization, Data curation, Resources, Writing – review & editing. **Caius Mustonen:** Conceptualization, Data curation, Writing – review & editing. **Till Demal:** Conceptualization, Data curation, Writing – review & editing. **Lenard Conradi:** Conceptualization, Data curation, Writing – review & editing. **Marek Pol:** Conceptualization, Data curation, Writing – review & editing. **Petr Kacer:** Conceptualization, Data curation, Writing – review & editing. **Angelo M. Dell’Aquila:** Conceptualization, Data curation, Writing – review & editing. **Konrad Wisniewski:** Conceptualization, Data curation, Writing – review & editing. **Igor Vendramin:** Conceptualization, Data curation, Writing – review & editing. **Daniela Piani:** Conceptualization, Data curation, Writing – review & editing. **Luisa Ferrante:** Conceptualization, Data curation, Writing – review & editing. **Timo Mäkikallio:** Conceptualization, Data curation, Writing – review & editing. **Eduard Quintana:** Conceptualization, Data curation, Writing – review & editing. **Robert Pruna-Guillen:** Conceptualization, Data curation, Writing – review & editing. **Antonio Fiore:** Conceptualization, Data curation, Writing – review & editing. **Thierry Folliguet:** Conceptualization, Data curation, Writing – review & editing. **Giovanni Mariscalco:** Conceptualization, Data curation, Writing – review & editing. **Metesh Acharya:** Conceptualization, Data curation, Writing – review & editing. **Mark Field:** Conceptualization, Data curation, Writing – review & editing. **Manoj Kuduvalli:** Conceptualization, Data curation, Writing – review & editing. **Francesco Onorati:** Conceptualization, Data curation, Writing – review & editing. **Cecilia Rossetti:** Conceptualization, Data curation, Writing – review & editing. **Sebastien Gerelli:** Conceptualization, Data curation, Writing – review & editing. **Dario Di Perna:** Conceptualization, Data curation, Writing – review & editing. **Enzo Mazzaro:** Conceptualization, Data curation, Writing – review & editing. **Angel G. Pinto:** Conceptualization, Data curation, Writing – review & editing. **Javier Rodriguez Lega:** Conceptualization, Data curation, Writing – review & editing. **Mauro Rinaldi:** Conceptualization, Data curation, Writing – review & editing.

## Declaration of competing interest

The authors declare the following financial interests/personal relationships which may be considered as potential competing interests:Fausto Biancari reports financial support was provided by 10.13039/501100005633Finnish Foundation for Cardiovascular Research. Fausto Biancari reports financial support was provided by Sigrid Jusélius Foundation.
